# PathBinder – text empirics and automatic extraction of biomolecular interactions

**DOI:** 10.1186/1471-2105-10-S11-S18

**Published:** 2009-10-08

**Authors:** Lifeng Zhang, Daniel Berleant, Jing Ding, Tuan Cao, Eve Syrkin Wurtele

**Affiliations:** 1 Iowa State University, Ames, Iowa, USA; 2 University of Arkansas at Little Rock, Little Rock, Arkansas, USA; 3 Ohio State University Medical Center, Columbus, Ohio, USA

## Abstract

**Motivation:**

The increasingly large amount of free, online biological text makes automatic interaction extraction correspondingly attractive. Machine learning is one strategy that works by uncovering and using useful properties that are implicit in the text. However these properties are usually not reported in the literature explicitly. By investigating specific properties of biological text passages in this paper, we aim to facilitate an alternative strategy, the use of *text empirics*, to support mining of biomedical texts for biomolecular interactions. We report on our application of this approach, and also report some empirical findings about an important class of passages. These may be useful to others who may also wish to use the empirical properties we describe.

**Results:**

We manually analyzed syntactic and semantic properties of sentences likely to describe interactions between biomolecules. The resulting empirical data were used to design an algorithm for the PathBinder system to extract biomolecular interactions from texts. PathBinder searches PubMed for sentences describing interactions between two given biomolecules. PathBinder then uses probabilistic methods to combine evidence from multiple relevant sentences in PubMed to assess the relative likelihood of interaction between two arbitrary biomolecules. A biomolecular interaction network was constructed based on those likelihoods.

**Conclusion:**

The text empirics approach used here supports computationally friendly, performance competitive, automatic extraction of biomolecular interactions from texts.

**Availability:**

http://www.metnetdb.org/pathbinder.

## Introduction

Increasingly large collections of gene sequence and expression data continue to appear. Biomolecular interaction databases are one kind of collection and are useful for such tasks as understanding biological processes [1], extrapolating knowledge about organisms to make predictions about other organisms as in BioCyc [2], and serving as components of larger resources like MetNet [3]. A database can be populated through expert curation, like MIPS [4] and KEGG [5]. In particular, extracting interactions from literature by expert curation has attracted considerable attention. Efforts include the Database of Interacting Proteins [6], BIND [7] and BioCyc. Manual methods are costly, however, so work has increasingly focused on automatic interaction extraction from scientific literature based on text mining technology. Extracted interactions can help researchers use knowledge buried in the literature and can even be used to construct interaction databases automatically.

Analysis of passages containing biological term co-occurrences or tri-occurrences enables the extraction of relations among biological entities. There are different methods of automatically extracting interactions between pairs of biomolecules from the literature, including readily implemented co-occurrence based methods [8], corpus-based statistical methods, template matching methods, and natural language processing [9].

### Natural language processing

Santos et al. [10], Natarajan et al. [11], Fundel et al. [12] and Rinaldi et al. [13] used full parsing to verify matches to predefined rules about descriptions of relations. Miyao et al. [14] used different natural language parsing tools to extract interactions and compared the results. Giles and Wren [15] applied full parsing in conjunction with a support vector machine (SVM) to extract the directions of interactions, since in a pair of interacting entities one tends to be the cause of an effect on the other. However full parsing is computationally expensive and relatively slow, subject to ambiguous parse results, and will only be a partial solution to the natural language processing (NLP) problem which includes semantic and other issues.

Yakushiji et al. [16] built a term recognizer to identify multi-word terms and a shallow parser to reduce lexical ambiguity. Then, they applied full parses over the preprocessed sentences. From the full parses, domain-specific knowledge including a set of target verbs and mapping rules provided by domain specialists was used to construct frame representations of interactions. Another example, GENIES [17], extracted semantic patterns by observing typical semantic and syntactic co-occurrence patterns in a sample corpus using semantic relationship categories and biological objects. It fully parsed sentences and outputted a frame structure when pattern matching was successful. GIS [18] and GIFT [19] also matched sentences to predefined interaction description patterns to identify the interactions.

There are different degrees of NLP, of course, and one way to make NLP more practical with large amounts of text is to use shallower analyses. Chilibot [20] takes this approach, using POS tagging followed by shallow parsing to extract interactions from MEDLINE and support a search engine for interactions in MEDLINE.

### Template matching

Template matching approaches form another and typically computationally more tractable strategy. A sentence, abstract or parsed result is matched against predefined *patterns *associated with interactions [21]. A pattern is a partial specification of words and locations in a passage, such as <biomolecule1 verb "the" verb "of" biomolecule2 "into">. The template term 'biomolecule' in such a pattern might match, for example, any molecule synthesized by living organisms. The matching process can involve a simple match using shallow parsing to identify terms meeting category or other constraints, or a complicated full parsing that analyzes the syntactic structure of the passage before matching against parse result templates.

Although pattern-matching can yield relatively high precision because patterns may be derived from existing sentences describing interactions, recall may be limited because it is not possible to manually describe all possible patterns of biomolecular interaction descriptions [22]. Therefore some interaction descriptions will not match the manually derived patterns, so some interactions will not be extracted by the template approach. For example, MedScan [23] obtained a recall of 21% with relatively restrictive templates, while Koike et al. [24] achieved 54% with more unconstraining, inclusive templates that assumed some syntactic analysis.

### Term occurrence

Term occurrence based approaches can avoid the recall issue just noted. Marcotte et al. [25] identified discriminating words based on a training set of 260 MEDLINE abstracts describing yeast protein interactions, based on differences in frequencies of occurrence of those discriminating words. They used the probabilities of each word's appearance in documents describing interactions to train Naïve Bayesian classifiers to score a document and judge whether the document describes an interaction.

A direct approach to identifying an interaction is to find co-occurrences of two biomolecules in the literature. Dragon Plant Biology Explorer (DPBE) [26] parses documents provided by users using this type of co-occurrence criterion and displays the results in, among other forms, a network of interactions. Albert et al. [27] applied co-occurrence extraction to create a protein interaction database for nuclear receptors, then post-processed this database by manual curation to delete false interactions. PDQ Wizard [28] and Hofmann and Schomburg [29] also used co-occurrences and a subsequent filtering stage to extract interactions between biomolecules.

The iHOP system (e.g. [30]) converts MEDLINE into a navigable hyperlinked resource by extracting sentences from it that contain biomolecules and annotating them with hyperlinks from the biomolecular and interaction terms to related sentences. A Web-based interface provides flexible access to this resource. This and similar systems extract sentences that appear to provide evidence for biomolecular interactions from the literature, but do not analyze this evidence further for probabilities of interaction based on empirical investigations of sets of related sentences. This motivates the current work, which fills that gap.

Wren and Garner [31] assigned a weight 1 - *r*^*n *^to the potential relationship between co-occurring terms, where *n *is the number of times they co-occur and *r *is one value when the co-occurrence is in a sentence and another value, 0.58, when the co-occurrence is in an abstract but not the same sentence. Ding et al. [8] also reported 0.58 for abstracts, but found a value for sentences different from Wren and Garner's.

Because co-occurrence based methods are relatively simple they cannot, in theory, match the potential performance of methods that incorporate information obtained by additional computation such as sentence parsing. However, they are computationally simpler and faster. NLP can get more out of text than co-occurrence based methods, while empirical facts derived from empirical analyses can provide heuristic guidance to NLP-based methods to enhance computational speed and help resolve ambiguities that arise. Thus, automated text analysis using a hybrid of both empirical facts about texts and deeper NLP-based analyses is expected to do better than either method alone. As an example, Zhou and He [32] used a machine learning method to estimate probabilities that help parse a document.

## Methods and analysis

This paper seeks to advance understanding about the properties of biomedical texts and to apply this knowledge to automatic identification of biomolecular interactions. Properties of texts were identified empirically (i.e. by examining actual sentences) and used to evaluate the probability that a given sentence describes an interaction between a specific biomolecule pair. A major issue in evaluating such extracted interactions is how to specify a good ranking policy. Such a policy would facilitate assessment of putative interactions.

By *empirical *we refer to knowledge about text properties derived from "experience or observation" [33]. Our observations are derived by manually examining corpora, and tabulating and analyzing the passages therein. This is distinguished from other common approaches to extracting knowledge from text such as Natural Language Processing, which deduces knowledge from passages based on syntactic and semantic rules, and Machine Learning (ML). Machine learning offers a corpus-based, statistical approach like the text empirics approach, but differs in that with ML, text properties are found automatically by a computer. This has the following shortcomings compared to using text empirics.

1) Classification rule sets (typically arranged in decision trees) derived by ML usually include uninteresting junk mixed in. As a result,

2) the rules derived by ML are typically omitted from publications, in favor of conclusions about the parameters of the ML process itself. As a result,

3) the outcome of ML can be harder to apply than the results of an empirical text analysis, since ML-derived knowledge tends to be less readily available in a directly usable form, while text empirics-derived results must necessarily be disseminated in an explicit form readily used by software designers.

Our software, PathBinder, extracts ranked interactions and provides query functions. Users can search for sentences describing interactions in MEDLINE by providing a pair of biomolecules. The entire comprehensive MEDLINE collection is searched for these sentences and the returned sentences can be ranked by their calculated likelihood of describing an interaction between the biomolecules. PathBinder can combine the evidence from multiple sentences to assess the relative likelihood of an interaction between two given biomolecules, and construct a biomolecular interaction network from MEDLINE automatically.

We chose MEDLINE as the repository to analyze. Much text mining research uses the MEDLINE collection http://www.nlm.nih.gov/pubs/factsheets/medline.html. MEDLINE contains approximately 18 million citation records to articles in the life sciences. A query interface, PubMed http://www.ncbi.nlm.nih.gov/pubmed/, enables users to search the records, and the Entrez Programming Utilities http://www.ncbi.nlm.nih.gov/entrez/query/static/eutils_help.html lets developers write software to access these data. While these records may not completely reflect the idea that an article tries to communicate, they usually contain the abstract and thus the most important information that the authors wish to convey. Using MEDLINE, Ding et al. [8] showed that sentences are useful text units for automatically extracting interactions. Therefore we collected sentences containing biomolecule co-occurrences to analyze as the basis of this work.

To extract an interaction we require a sentence to contain two biomolecules of interest. However such a sentence does not necessarily describe an interaction. For example, the sentence

"Both A and B can bind to C."

does not describe an interaction between A and B, even though it describes interactions between A and C, and between B and C. Our hypothesis is that we can find properties of sentences from the MEDLINE collection that can support automatic interaction extraction. The first goal is therefore to advance understanding of relevant sentence properties. The second and related goal is to better understand properties of interaction-indicating terms (IITs). The third goal is to use results of the first and second goals to predict whether a sentence describes an interaction. The fourth goal is to scale up by generating and evaluating a database of biomolecular interactions.

By analyzing typical passages from MEDLINE it is possible to focus on those goals by empirically investigating certain questions such as the following.

1) How can the presence of IITs (interaction-indicating terms) be used to infer the type of interaction between two specific biomolecules?

2) If *p*_phrase _is the likelihood that biomolecules co-occurring in the same phrase are described by the phrase as interacting, how does *p*_phrase _differ from *p*_sentence_, the analogous situation where they are in different phrases of the same sentence?

3) How does the order of appearance of three important words, two biomolecules and an IIT, in a phrase or sentence affect the probability that the biomolecules are described as interacting?

4) How do properties of IITs occurring near two biomolecule names, such as their identities, inflections, roots, and semantic categories, affect the probability that they help describe an interaction between the biomolecules?

For questions 1–4, we collected 303 MEDLINE abstracts and extracted 664 sentences, based on ten queries to PubMed. Each query consisted of two biomolecule names known to interact, and was elicited from biologists to be typical of the kinds of queries biologists are likely to make. Some further details about this corpus appear in Ding et al. (2002 [8]), and a list of the abstracts in the corpus may be downloaded from http://ifsc.ualr.edu/jdberleant/IEPA/IEPA.htm. Each sentence was manually analyzed with respect to the properties related to questions 1–4 above and tagged as to whether or not it described an interaction between the two query biomolecules.

To support the accurate description of passage properties for interaction extraction, we use the definitions shown in Table 1.

**Table 1:** Definitions used in text analyses.

Term	Definition
*sentence*	Either an article title, or a word sequence beginning with a capital letter and ending with a period.
*phrase*	A word sequence that occurs inside a *sentence*, and begins and ends with: , | ; | : | . | <the beginning of the sentence> | <whitespace>-<whitespace> | (|).
*IIT*	*Interaction-indicating term. *A word, often a verb, that can describe an interaction between two biomolecules.

We have manually created a list of IITs based on reading several hundred MEDLINE abstracts. For example, *activate*, *activation*, etc., can describe an interaction between two biomolecules, as in "the activation of A by B."

The results for questions 1 and 2 (Table 2) indicate that the probability an interaction is described when two biomolecules co-occur in a phrase is higher than when they are in different phrases in a sentence (67% vs. 33%). Secondly, if an IIT appears with the two biomolecules, the probability that an interaction is described is higher than without an IIT present (55% vs. 7.99% and 71% vs. 0%). These two comparisons are statistically significant (p < 0.001, χ^2 ^test).

**Table 2:** Biomolecule co-occurrences in sentences and phrases, with and without IITs.

	# (%) that describe the interaction	Total number
**Sentences where two biomolecules tri-occur with at least one IIT**	331 (55%)	606
**Sentences where two biomolecules co-occur without any IIT**	3 (7.9%)	38
**All sentences where two biomolecules co-occur**	334 (52%)	644
**Phrases where two biomolecules tri-occur with at least one IIT**	236 (71%)	334
**Phrases where two biomolecules co-occur without any IIT**	0 (0%)	17
**All phrases where two biomolecules co-occur**	236 (67%)	351
**Sentence co-occurrences not in phrases**	98 (33%)	293

For question (3), we investigated how an IIT present between the two biomolecules differs from when an IIT is present but not between the biomolecules. The results are shown in Table 3.

**Table 3:** Percentages of sentences and phrases describing interactions (i.e., precisions), by IIT location.

	IIT intervening	IIT elsewhere in sentence	IIT in either place
**Phrases in which two biomolecules co-occur**	63%	24%	45%
**Sentence co-occurrences that are not also phrase co-occurrences**	30%	9.1%	21%
**Both phrase and sentence co-occurrences**	48%	17%	34%
**Percent of interaction descriptions**	77%	23%	100%

Table 3 shows that the presence of an IIT intervening between the two biomolecule names is associated with relatively high likelihood that an interaction is described. Consequently, for descriptions in which one or more IIT was present, most (77%) had an IIT between the biomolecule names.

For question (4), we collected a new set of 320 sentences from the results of 10 queries to PubMed. The queries were picked by biologists to represent typical interests. In addition, these 320 sentences were required to contain at least one IIT, thus permitting us to analyze IIT properties. The queries were *nitrite & xanthine*, *pyruvate dehydrogenase & phosphofructokinase*, *indole acetic acid & starch*, *glucose & starch*, *glucose-6-p & starch*, *carotenoid & IPP*, *cre & cytokinin*, *acetyl-CoA & leucine*, *glucose & pyruvate*, and *ATP & myosin*.

Syntactic and semantic categories of the IITs in each sentence were recorded along with whether an interaction was described between the pair of biomolecules specified by the query. From these data, we investigated the possibility that IIT *form *(noun, adjective, adverb, present, present continuous and past/perfect) and *semantic category *(association, modification, negative regulation, positive regulation, transportation, transcription, create, and vague) can be used as evidence for mining interactions from text. 'Vague' was used as the category when an IIT could not be clearly placed in one of the other categories. The past and perfect forms of IITs are sometimes the same, and the frequency of the perfect form is low, so we did not distinguish between them.

The noun form and the 'modification' category appeared more often than other forms and categories in sentences describing interactions. However this combination also appeared in more sentences overall than others. More details appear in Tables 4 and 5, which give the percentages of sentences and phrases describing interactions between two given biomolecules broken out by IIT forms and categories. Note that some IITs have the same spelling for both the noun and present tense forms. We can manually differentiate them but to use those results in automatic methods would require parsing at least to the extent of POS tagging.

**Table 4:** Data on likelihoods that sentences describe interactions when they contain biomolecule co-occurrences that are not in the same phrase.

Forms	# (%) of sentences describing interactions	Total sentences
Noun	141 (59%)	237
Adjective	9 (45%)	20
Present	50 (66%)	76
-ing	35 (51%)	69
Past/Perfect	77 (55%)	141
**Categories**		
Association	60 (67%)	89
Modification	80 (66%)	121
Negative regulation	33 (39%)	84
Positive regulation	47 (42%)	112
Transportation	14 (67%)	21
Transcription	5 (71%)	7
Create	63 (66%)	96
Vague	41 (54%)	76

Comparing the 13 rows in Table 4 and the corresponding rows in Table 5, a phrase containing a biomolecule pair has a higher probability of describing an interaction than a sentence containing the pair not within a single phrase in that sentence (*p *< 0.005, *t *test on the 13 *z *values).

**Table 5:** Data on likelihoods that phrases containing biomolecule co-occurrences describe interactions, by interaction-indicating term form and category.

Forms	# (%) phrases describing interactions	Total phrases
Noun	97 (66%)	148
Adjective	3 (43%)	7
Present	31 (74%)	42
-ing	16 (55%)	29
Past/Perfect	56 (65%)	86
Association	41 (75%)	55
Modification	60 (78%)	77
Negative regulation	24 (49%)	49
Positive regulation	30 (52%)	58
Transportation	7 (54%)	13
Transcription	2 (100%)	2
Create	37 (73%)	51
Vague	31 (65%)	48

PathBinder combines the evidence provided by various attributes of a sentence by multiplying odds for each attribute to calculate the overall probability that the sentence describes the putative interaction (e.g.Manning et al 2008 sections 11.1, 11.3 [34]; Davis 1990, pp. 128-130 [35]). The formula used (Dickerson et al 2005 section 2.3.3 [36,37]) is *O*(*h*|*f*_1_,..., *f*_*n*_) = *O*(*h|f*_1_)*O*(*h|f*_2_)...*O*(*h|f*_*n*_)/*O*(*h*)^*n*-1 ^which expresses the odds of hypothesis *h *(in this case that a given passage describes an interaction between given biomolecules) given *n *items of evidence in terms of a default odds *O*(*h*) modeling the entire corpus, and *O*(*h|f*_*k*_), *k *= 1,..., *n*, which are the odds of the hypothesis given evidence item (in this case, sentence feature or attribute) *k*. Odds convert to probability by *p *= odds/(1+odds), so that for example odds of flipping heads instead of tails is H:T = 1:1 = 1, so *p *= 1/(1+1) = 0.5 as expected.

Calculating probabilities sentence by sentence permits ranking sentences based on those probability scores. However, when the goal is to obtain the overall probability of an interaction, we must also combine the evidence provided by multiple sentences containing the same biomolecule co-occurrence. This is explained next.

### Combining evidence from multiple passages

A sentence can be given a likelihood of describing an interaction based on its containing a co-occurrence, whether in a phrase, or in the sentence but across phrases. Multiple sentences containing the same co-occurrence often exist in MEDLINE, so to extract interactions from MEDLINE we would like to combine the multiple sources of evidence constituted by the multiple sentences. This can be done probabilistically as follows. Let *p *be the probability that a sentence describes an interaction. Then *q *= 1-*p *is the probability that it does not. Given *n *such independent sentences, and assuming for a moment that probability *p *is the same for all sentences, then *q*^*n *^would be the probability that none of them describe an interaction, thus 1-*q*^*n *^the probability that at least one does. Since *q *= 1-*p*, the formula for the probability of an interaction between a pair of biomolecules being described within *n *relevant sentences is 1-(1-*p*)^*n*^.

In the more typical case of *n *sentences each with its own value *p*_*i*_, *i *= 1,..., *n *for the probability that it describes an interaction, the formula generalizes to:(1)

assuming the sentences provide independent evidence, an assumption commonly made and found to lead to useful results though in general incorrect.

It is reasonable to ask if the value of *n *should be constrained. Some new interactions may be mentioned only in the most recent publications, limiting the number of publications describing these interactions. Thus, particularly for a recent discovery, the fact that only a few sentences exist containing two given biomolecules might not suggest lack of interaction. Therefore, we also assessed two variant methods for estimating the probability of an interaction between two biomolecules. These are as follows:

• *Best 5*: use the *average *of the scores of the top 5 sentences, those having the highest probability of describing an interaction between the two biomolecules: *p*(interaction) = (*p*_1_+*p*_2_+*p*_3_+*p*_4_+*p*_5_)/5.

• *Best 2*: use the *average *of the scores of the top 2 sentences: *p*(interaction) = (*p*_1_+*p*_2_)/2.

Formula (1) we will call the *All *method. For the *Best 2 *and *Best 5 *methods, if a biomolecule pair co-occurs in fewer than 2 or 5 sentences, 0 was used for the missing probabilities to reach 2 or 5 terms in their formulas.

With these 3 evidence combination methods, given a list of biomolecule pairs we can process MEDLINE to extract biomolecular interactions and construct an interaction network. The biomolecules are the vertices in this network, and if two biomolecules are found to interact, there is an edge between their vertices. We obtained the biomolecule name list from an existing database about genome-wide plant mRNA, protein, and metabolite data, MetNetDB (http://metnet.vrac.iastate.edu/MetNet_db.htm, [3]). This database focuses especially on *Arabidopsis *and soy. We created an interaction network from this database to demonstrate our system.

**Figure 1:**
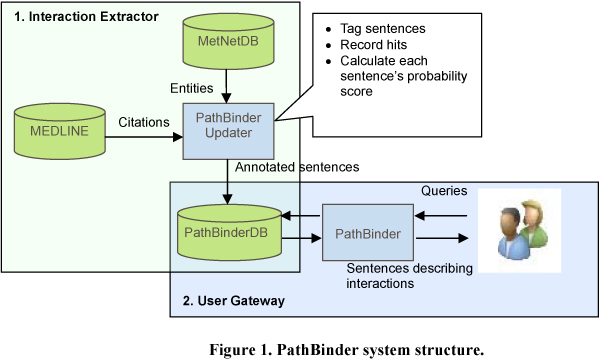
**PathBinder system structure**.

Any two biomolecules in the database can be checked to see if they interact. For each such pair any sentences where the biomolecule pair co-occurs can be collected and analyzed to estimate the probability that the corpus describes them as interacting. However, checking all pairs is computationally inefficient because there are about 2*10^6 ^biomolecule records in the database, hence about 4*10^12 ^pairs. Instead, we scanned sentences in MEDLINE one by one, identified biomolecule pairs in the sentences, recorded the probability score that each sentence gives to its pairs and finally generate the network using the *All, Best 5 *and *Best 2 *combination methods on the sentences for each pair. The overall structure of the system is shown in Figure 1.

There are two main parts.

1. *Interaction Extractor*.

a. The system examines each sentence in MEDLINE for keywords (biomolecules, IITs, & cellular locations) stored in MetNetDB, tags them and stores the tagged sentences into the PathBinder system database, PathBinderDB.

b. When scanning each sentence, the system determines the interaction likelihood for the biomolecule co-occurrence of interest inside the sentence and combines the scores of multiple sentences containing the pair using *All*, *Best 5*, and *Best 2*. The database has two tables for biomolecules, one for their appearances in MEDLINE records and one for entity names recognized by biologists but which might not appear in MEDLINE records under those names. These tables were imported from the MetNet system (Wurtele et al. 2007 [3]). When combining the scores, we first calculated the score for the actual co-occurring pair, then found the entity names in the database corresponding to the co-occurring terms appearing in the text, and finally calculated the composite score for the pair of entity names based on the set of sentences containing co-occurrences of other terms associated with those entity names.

2. *User Gateway*. PathBinder is the user portal to PathBinderDB. PathBinder serves as a query gateway to interaction descriptions stored in PathBinderDB. Users can provide two biomolecules to PathBinder, which will access PathBinderDB and return all sentences in which the two biomolecules appear. It calculates a probability score for each returned sentence, ranks sentences based on their scores, and then shows them to the user. On the other hand, if a user provides just one biomolecule, PathBinder returns a list of other biomolecules potentially interacting with it.

## Results and testing

### Evaluating sentences as interaction descriptions

**Figure 2:**
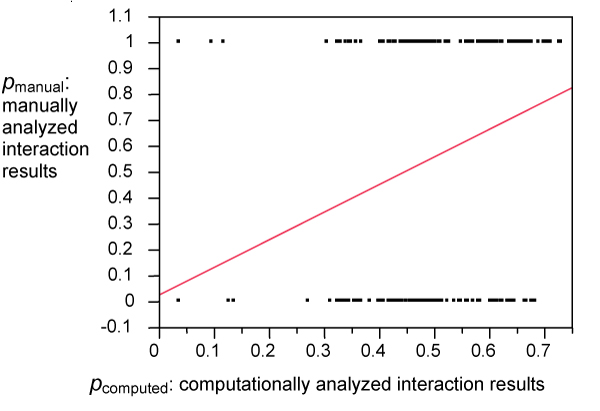
**Linear regression results: computation vs. manual analysis (theoretical ideal: *p*_manual _= *p*_computed_)**. Note that the 320 manually determined data points all have probability values of 0 or 1 (either they describe an interaction or not), so many of them overlap in the graph.

We began with the test corpus of 320 sentences described earlier, for which we computed 320 probability estimates for the likelihood that they described an interaction between a given biomolecule pair. We also manually judged whether each sentence actually does describe an interaction between the queried biomolecule pair, recording 1 if so, or 0 if not, in order to facilitate doing a linear regression to fit the 320 computed likelihoods to the 320 corresponding manual data. If the probability that a sentence describes an interaction is computed accurately, then for a set of sentences with the same computed probability of describing the interaction (e.g., 0.75), that probability is also the expected fraction of those sentences manually found to actually describe the interaction. For example, given a set of sentences each computed to describe an interaction with probability *p *= 0.75, the statistically expected fraction of them to, in fact, describe an interaction would also be 0.75 (75%), if the computed probability was accurate. Therefore, we can test the accuracy of the computed probabilities by checking how close the linear regression result is to the line *y *= *x *(or for axes labeled as in Figure 2, *p*_manual _= *p*_computed_). We consider the actual regression result next.

The regression line shown in Figure 2 is not precisely *p*_manual _= *p*_computed_, but is fairly close:(2)

To make our computed probabilities more accurately reflect manually determined reality (i.e., give a regression line of *y *= *x*), we can adjust them by defining a *p*_adjusted_:(3)

It is no accident that Eqs. (2) and (3) are so similar: showing *p*_adjusted _on the *x *axis will then give a regression line of *y *= *x *or, in the present case, *p*_manual _= *p*_adjusted_, as desired.

We applied (3) in PathBinder, so that for each sentence *s*, a computed probability score *p*_computed_(*s*), is calculated and then adjusted to give a probability score *p*_adjusted_(*s*) for the probability that it describes an interaction between two given biomolecules.

The discrepancy between *p*_computed _and *p*_manual _has two possible causes. First, it can simply be a statistical artifact of noisy data. Second, the computational model underlying *p*_computed _might represent reality imperfectly, as models in general often do, and as probabilistic models in particular often do due to implicit independence assumptions that only approximately hold.

To help determine the cause here, and thus test the validity of the *p*_*adjusted *_calculation, we collected a test set of 600 sentences. Of these, 123 contained the 10 biomolecule pairs from among the 10 we used to create the training corpus, but were not already in the 320 sentence experimental set. To get the remaining 477, we collected sentences with *p*_*adjusted *_values of 0, 0.1 ± 0.01, 0.2 ± 0.02, 0.3 ± 0.03, 0.4 ± 0.04, 0.5 ± 0.05, 0.6 ± 0.06, 0.7 ± 0.07 and 0.739 ± 0.07 (the *p*_*adjusted *_computation gives results up to about 0.739). About 50 sentences for each of those values were collected from search results using the new pairs: *ethanol & acetaldehyde, acetyl-CoA & NADH, dynamin & GTP, adenylate cyclase & ATP*, and *ATP & creatine*.

**Figure 3:**
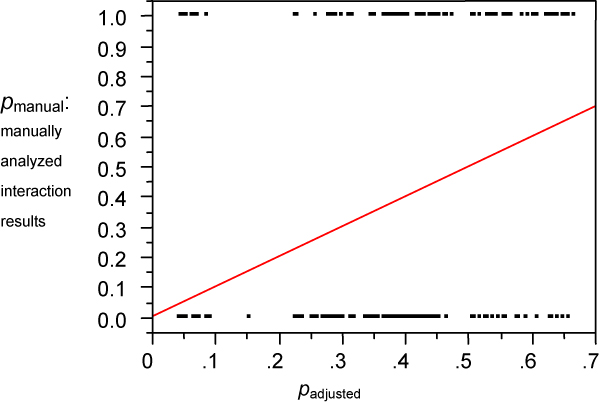
**The linear regression results for the test set of 600 sentences**. Note that the 600 manually determined data points often overlap because (i) they all have a height of either 0 or 1, as they were all manually determined to describe an interaction (1), or not (0), and (ii) most of them have horizontal axis values very close to 0, 0.1, 0.2, 0.3, 0.4, 0.5, 0.6, or 0.7.

For each of the 600 test sentences, whether it really described the interaction was judged manually and recorded as 0 (no) or 1 (yes). Then we did a linear regression on the test set (Figure 3) as was done earlier in Figure 2.

The regression line we get is(4)

which is very close to the ideal of *y *= *x*. Thus PathBinder's calculation of *p*_adjusted _is justified by test set B.

### Combining evidence across sentences to create an interaction network

**Figure 4:**
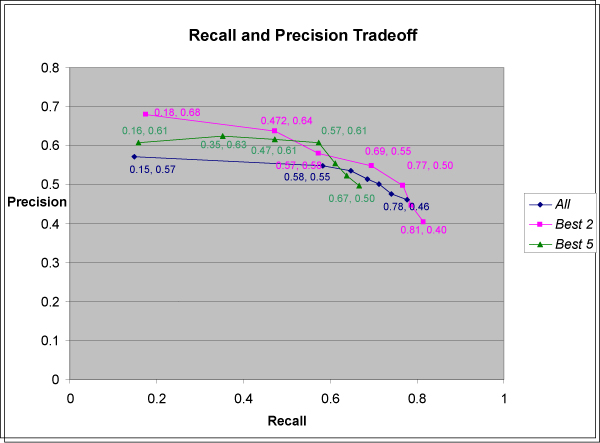
**Recalls and precisions of the three methods for combining evidence from multiple sentences**.

Equation (3) is used to evaluate the likelihood that *each *sentence describes an interaction. As mentioned earlier, we combine evidence from *multiple *sentences to evaluate the likelihood that a pair of biomolecules interacts using Equation (1) or the *All *method, and the *Best 2 *and *Best 5 *methods. The result is an interaction network of thousands of biomolecules and the interaction relationships among them. The key information retrieval measures of precision and recall were used to compare *All*, *Best 5*, and *Best 2*. Some key results are shown in Figure 4.

To determine the precisions in Figure 4, we randomly sampled a set of 400 pairs of biomolecules co-occurring in MEDLINE from the previously generated interaction network. The sentences for each pair were each evaluated by the three methods (*All*, *Best 5 *&* Best 2*), and the resulting computationally estimated probabilities of interaction were recorded for each pair. The 400 pairs were also manually analyzed to see whether they do in fact interact. One hundred eight of them did interact. The overall precision was thus 108/400 = 0.27 for this random set. More importantly, we calculated the precisions analogously for 7 subsets of the 400 pairs meeting 7 different thresholds for interaction probability. This was done separately for *All*, *Best 5*, and *Best 2 *(making 7*3 = 21 subsets). Thus each subset was associated with a threshold, a calculation method, a precision, and a recall which was the fraction of the 108 interacting pairs meeting the threshold using the calculation method. The overall recall for the whole set is necessarily 1.

Some aspects of Figure 4 are worth considering further. For the *All *method, the leftmost data point refers to co-occurrences with a calculated interaction probability of 1. Such a high value happens when there are a lot of sentences providing evidence. Combining that evidence using equation (1) leads to score values that are effectively 1 (for example, co-occurrences of "bilirubin" and "cytochrome P450" and their synonyms was computed to have a score of 1–10^-11^). We counted any score over 1–10^-6 ^as 1. This was therefore the most selective threshold for the *All *method and it occurred for 342,492 biomolecule pairs (for MEDLINE as of October 2008).

Unlike the *All *method, the *Best 5 *and *Best 2 *methods only look at average scores of sentences, so calculated probability scores tend to be lower for these methods than for the *All *method. Thus *Best 5 *and *Best 2 *permit score thresholds met by fewer than 342,492 pairs.

The curves in Fig. 4 are not always monotonic. For example, the first part of the *Best 5 *curve is not monotonic. The leftmost point on that curve, (0.16, 0.61) is based on the 28 pairs meeting or exceeding a threshold score value of 0.58, computed by the *Best 5 *method. This was the most selective threshold used to generate the curve. Yet the 62 pairs that met a lower threshold of 0.53 actually had a higher precision, giving point (0.35, 0.63) in Figure 4. One possible reason is noise from the limited data. Another possibility is that *Best 5 *actually does produce this effect for some reason.

**Figure 5:**
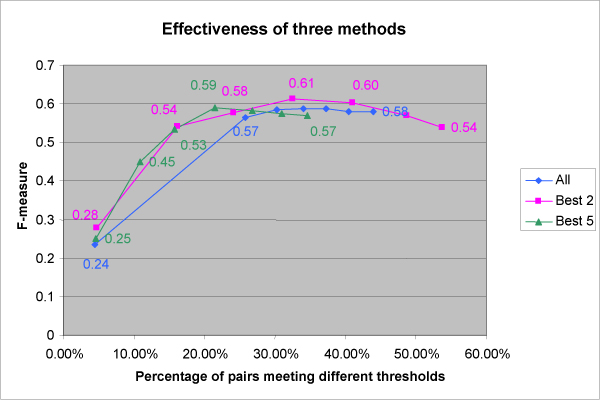
**Effectiveness (*F*-measure) comparison of the three methods**.

Recall and precision are often combined to get a single, composite measure of information retrieval quality called the effectiveness, or *F*-measure, of an information retrieval method: *F *= 2(recall*precision)/(recall+precision). Figure 5 shows the effectiveness for the three methods as a function of the size of the subset meeting a given threshold, with size expressed as a percentage of the full 400-member set.

For the *F *measure, the *Best 2 *method gave the highest peak value, for a threshold met by 137 pairs. For the full result interaction network, there are 1,646,337 pairs that meet that threshold.

### Use in PathBinder

**Figure 6:**
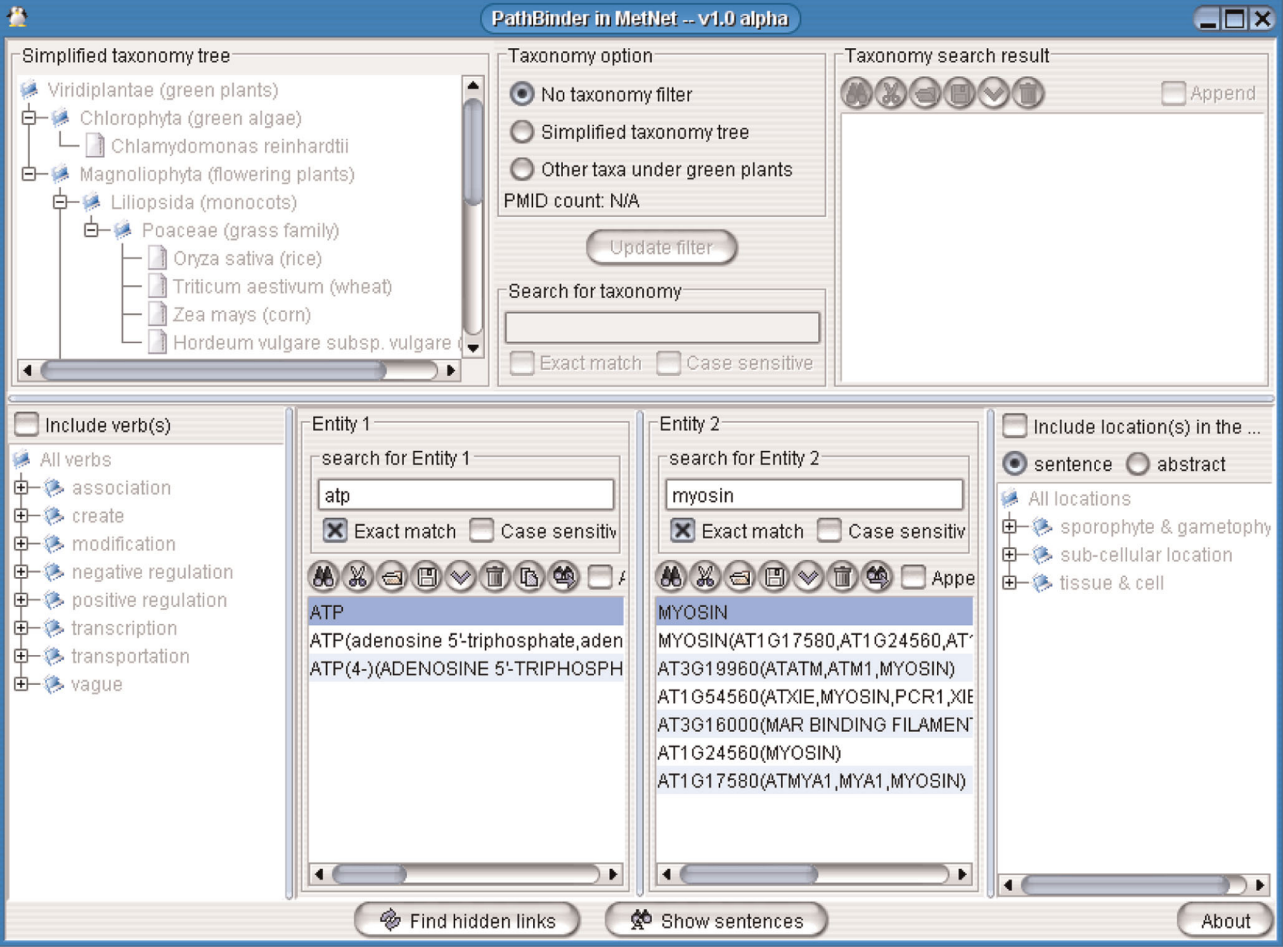
**PathBinder main screen**.

**Figure 7:**
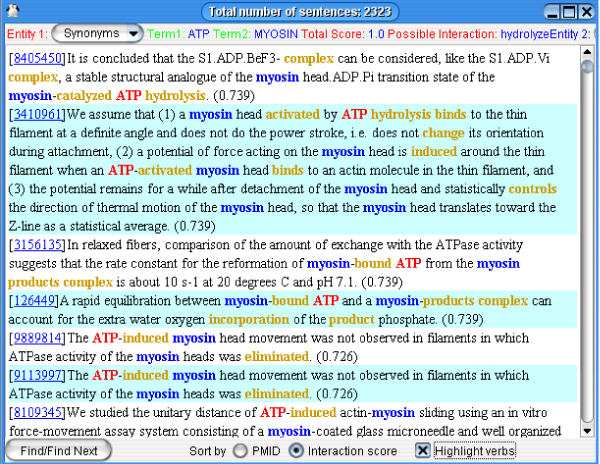
**PathBinder search results**.

Our technique has been applied in the PathBinder System, which provides a query gateway to users. If a user provides a biomolecule, PathBinder can find other biomolecules potentially interacting with it. Users can choose a biomolecule pair as a query for sentences describing interactions, as illustrated in Figure 6. Users can also specify more query conditions, like cellular locations (e.g., nucleus, mitochondrion, etc.), categories of IITs appearing with the co-occurring biomolecule names (e.g. association, modification, etc.), specific IITs appearing with a co-occurrence (e.g. bind, increase, etc.) and Linnaean taxonomic categories. All these data are obtained when processing MEDLINE and were pre-recorded in the database. Once Pathbinder gets a query, it will search for all sentences satisfying the query and display them in a new window, as in Figure 7. It can order the result sentences by PMID or (as in Fig. 7) by their estimated probability of describing an interaction between the biomolecules. Users can click the PMID to read the PubMed record containing the sentence directly on the PubMed Web site.

## Discussion

As explained earlier, we calculate a rather precise probability estimate that a sentence describes an interaction between a given biomolecule pair. However, this precision can be misleading. A typical problem is that an IIT describes the interaction of one biomolecule in the given pair with another biomolecule not in the pair, but the non-syntactic approach of PathBinder mistakenly concludes the interaction may be between the biomolecules of interest. For example, consider the sentence

*Sodium dichloroacetate increased ****glucose*** *oxidation and* ***pyruvate *** *oxidation in hearts from fed normal or alloxan-diabetic rats perfused with glucose and insulin. *[38]

The term "oxidation" is between the biomolecules "glucose" and "pyruvate" but it does not describe an interaction between them. PathBinder, however, gives a high score to this sentence anyway. Analyzing the syntactic structure of the sentence, as with full parsing or link grammar [39] would help solve this problem, but is computationally more expensive.

Another typical problem is that some IITs are not recognized. An unusual IIT might not be stored in our database and so would not be recognized. For example, consider the following sentence.

***GTP*** *-dependent twisting of* ***dynamin**** implicates constriction and tension in membrane fission. *[40]

If we try to find an interaction between GTP and dynamin, there is no obvious IIT describing their interaction. But the word "dependent" describes a relation between "GTP" and "twisting of dynamin," so that there is indeed an interaction described. However, neither "dependent" nor "twist" are currently used by the system as IITs and so this sentence gets too low a score.

Another problem occurs with biomolecules that are very common in MEDLINE. The chance that two of them co-occur in one sentence can be elevated even if they do not interact just because they are so common overall. Most sentences that they co-occur in might not get a high estimated probability of describing an interaction, but if even a small fraction of them do, the estimated probability of interaction can still be high. An example is "ATP" and "starch."

**Figure 8:**
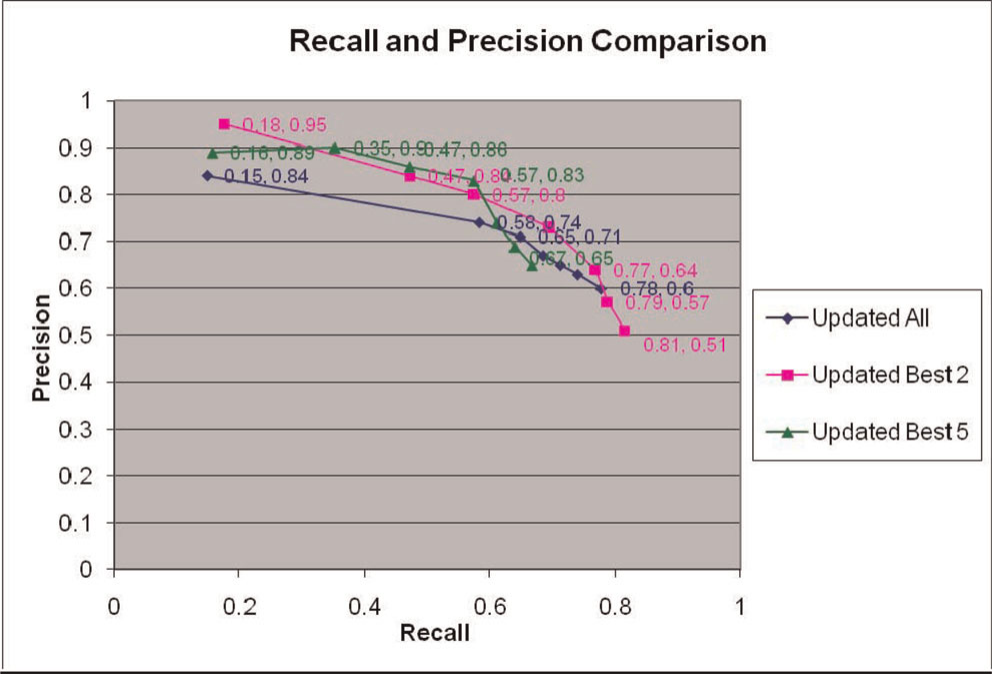
**Updated recalls and precisions of the three methods for combining evidence from multiple sentences**. Precisions are markedly improved when problematic "biomolecule names" are manually removed from consideration (compare this with Figure 4).

**Figure 9:**
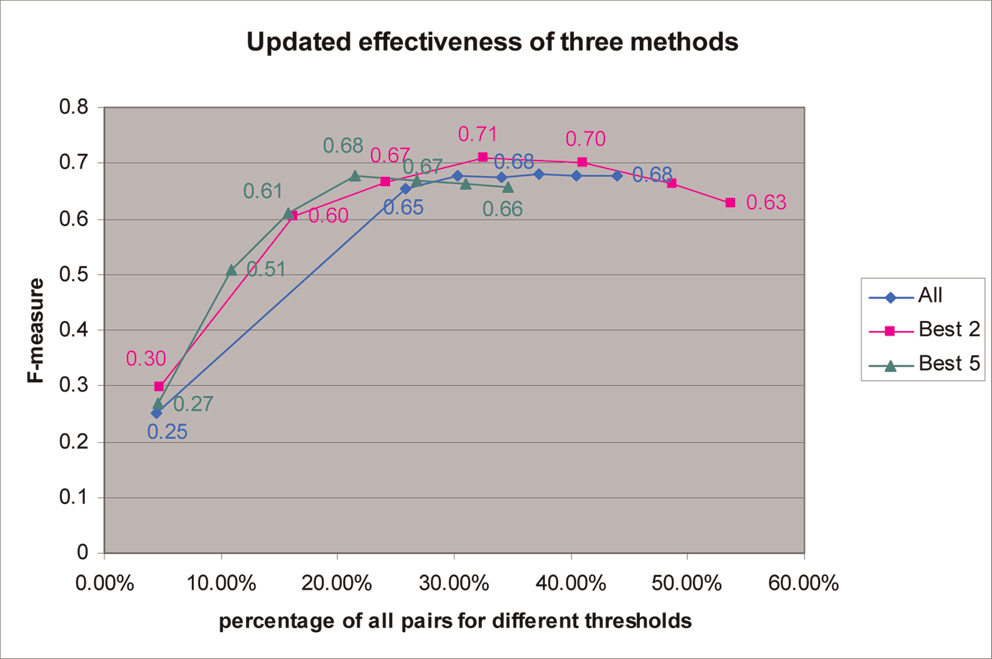
**Updated effectiveness comparison of the three methods**. Problematic "biomolecule names" are manually removed from consideration and effectivenesses increased compared to Figure 5.

A different problem in network construction is posed by biomolecules that look like common words in English. For example, since the word 'no' and the abbreviation of nitrous oxide have the same spelling, and the token "no" appears very often in MEDLINE, a naïve analysis will mistakenly conclude that nitrous oxide has interactions with thousands of biomolecules. In addition, some non-biomolecule terms tend to creep into lexicons of biomolecules, like "resistance" in our case. Such terms tend to then become members of invalid "interactions." In fact, if we eliminate the effects of words like "no" and "resistance," the precision of our results increases significantly, as shown in Figure 8. The effectiveness was in turn improved by the improved precision, as shown in Figure 9 (the recall stays the same in this test because no new interacting pairs appear).

Our precision results are higher than for some other interaction extraction applications. Our highest precision of 95% is among the best results for extracting interactions so far. NLP methods in principle should be capable of obtaining close to 100% precision and recall. Avoiding NLP, however, our system saves considerable time. Our results could be improved while retaining the computational efficiency of shallow methods by investigating and using empirics for more text features. Even when full NLP becomes available at some future time, easily computed text empirics will still have potential value as an ancillary evidence source that could improve and speed up NLP-based analyses.

## Conclusion

We created and developed algorithms to extract sentences describing interactions between biomolecules based on *text empirics*, that is, observed characteristics of textual passages. Using this approach we designed a software system that provides a service to users by extracting interaction descriptions from MEDLINE. The extracted sentences can be ranked by their estimated probability of describing an interaction between the two biomolecules. We compared the probability estimates to manually generated ("gold standard") data to test their accuracy. Results were close, as shown by Eq. (2), and nearly identical when estimates were linearly adjusted and then tested against a new test set. From MEDLINE, we extracted and created an interaction network which contains more than 300,000 probable interactions. The approach was demonstrated in a system architecture designed for human searchers. However the underlying text empirics results we offer here could be used by other researchers and system designers as well.

## List of abbreviations used

(NLP): Natural language processing; (ML): Machine Learning; (IITs): Interaction-indicating terms.

## Competing interests

The authors declare that they have no competing interests.

## Authors' contributions

LZ and DB analyzed the corpus and designed the algorithm. LZ, JD and TC developed the software. DB and ESW determined the goals, system architecture and usability design.
